# Short range smectic order driving long range nematic order: example of cuprates

**DOI:** 10.1038/srep19678

**Published:** 2016-01-27

**Authors:** R. S. Markiewicz, J. Lorenzana, G. Seibold, A. Bansil

**Affiliations:** 1 Physics Department, Northeastern University, Boston MA 02115, USA; 2 ISC-CNR and Dipartimento di Fisica, Università di Roma “La Sapienza”, P. Aldo Moro 2, 00185 Roma, Italy; 3 ISC-CNR, Via dei Taurini 19, I-00185 Roma, Italy; 4 Institut Für Physik, BTU Cottbus-Senftenberg, PBox 101344, 03013 Cottbus, Germany

## Abstract

We present a model for describing the combined presence of nematic and ‘smectic’ or stripe-like orders seen in recent scanning tunneling microscopy (STM) experiments on cuprates. The smectic order is treated as an electronic charge density wave with an associated Peierls distortion or a ‘Pomeranchuk wave’. This primary order is restricted to nanoscale domains by disorder effects, while the secondary coupling to strain generates the nematic order with a considerably longer range. A variety of experimental results are shown to be consistent with our theoretical predictions.

The interplay between nematicity and another fluctuating order parameter is a focus of current research on novel Fe-based as well as cuprate superconductors. In particular, it has been suggested^1^,^2^,^3^,^4^ that the transition (at temperature *T*_*s*_) from a tetragonal to an orthorhombic phase in the Fe-based superconductors is driven by fluctuating magnetic moments, which break the *Z*_4_ lattice symmetry above *T*_*s*_ due to frustration, before the antiferromagnetic order sets in at 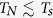. A related discussion has also been stimulated in the cuprates by the discovery of a coexisting short range (glassy) smectic order and a long range nematic order in Bi_2_Sr_2_CaCu_2_O_8_ (Bi2212)^5^. Ref. 6 shows that fluctuations of the nematic phase are strongly correlated with the smectic phase, and that this coupling can be described in terms of a Landau-Ginzburg (LG) theory developed originally for liquid crystal phases. In view of the great variety of models proposed for the CDW and stripe phases, LG type models have proven valuable for understanding model-independent properties of DW/stripe phases^1^,^7^. It is clear that a variety of correlated materials display the simultaneous occurrence of nematic and smectic orders, including systems with weak magnetic effects^8^, and it is important therefore to understand what generic mechanisms may be at play in this connection. Liquid crystals are composed of very anisotropic particles, so a primary nematic order parameter arises naturally. While acicular quasiparticles have been observed in the pnictides^9^ and predicted in cuprates^10^, in many theories the dominant instabilities are associated with Fermi surface nesting at finite q-vector, and the large nematic (q = 0) correlation length remains a puzzle. Here we explore this issue by invoking an LG type model to incorporate the effect of lattice strain.

We assume that the competing smectic order is a form of charge order (CO) and invoke a LG approach for incorporating effects of impurities, which act to break up the CO into small domains but still allow the development of large scale nematic distortions. The coupling of this nanoscale CO to strain is shown to provide an explanation for the smectic-nematic phase observed in STM experiments^5^. We emphasize that this specific mechanism of CO is not essential for our theory. Indeed, it may well be that different mechanisms are at work in various compounds, which might even be doping dependent. For example, in lanthanum cuprates CO is usually discussed in terms of ‘stripes,’^11^ which involve both charge-density wave (CDW) and spin-density wave (SDW) orders with the charge-enriched regions acting as antiphase domain walls for the magnetic order^12^,^13^,^14^,^15^,^16^,^17^,^18^,^19^. Within weak to intermediate coupling approximations of Hubbard type models such instabilities arise in the magnetic channel, and the stripe state results from the coupling between longitudinal magnetic and charge correlations deep in the instability regime. This mechanism accounts, for example, for the stripe state in La_2−*x*_Ba_*x*_CuO_4_ where in the underdoped region of the phase diagram both charge and magnetic order appear simultaneously when the temperature is lowered^20^.

Alternatively, one can approach the instability from the overdoped side where fluctuations prevent the formation of static magnetic order. Instead, the kinetic energy is reduced via strong correlations in such a way that any short range interaction could destabilize the system with respect to CO. Depending on the relative sizes of long-range Coulomb and short-range interaction, either a ‘frustrated phase-separation-instability’^21^ or a nesting induced instability toward an incommensurate CO state can be realized. Thus we have recently shown^22^ that CO in Bi2212 and Bi2201 is stabilized by nesting of the flat, nearly parallel antinodal (AN) FS sections near the (*π*, 0) point in the Brillouin zone^23^,^24^,^25^,^26^, and hence we refer to this order as AN nesting (ANN). This ANN phase breaks *C*_4_ symmetry and it thus also represents a quasi-one-dimensional CO state^27^. The resulting CO provides a good description of many properties of the modulated phase found in scanning tunneling microscopy (STM) studies of Ca_2_CuO_2_Cl_2_ (CCOC), Bi2212, and Bi_2_Sr_2_CuO_6_ (Bi2201)^24^,^25^,^26^,^27^,^28^,^29^,^30^, and CDW studies of YBCO^31^,^32^,^33^,^34^,^35^,^36^.

Perhaps the most puzzling feature of the CO phase is that it coexists with a *q* = 0 nematic order which breaks the symmetry of the unit cell, making the oxygens inequivalent, with a correlation length much larger than that of the CO. Such an anomalous behavior could be understood in the presence of charge disorder for two reasons. Firstly, disorder acts as a quenched random field for the CDW phases, which destroys the long-range order in two-dimensional systems, leaving behind an array of finite-sized clusters. Secondly, the associated lattice distortions can couple to lattice strain, and we will demonstrate that since the strain coupling is long-range, it can lead to *q* = 0 strain domains much larger than the initial stripe domains, in good agreement with experiments.

## Formalism

It is important to recognize at the outset that the secondary coupling to strain should arise naturally in any model in which stripes are coupled to lattice distortions. Note here that the hopping parameter *t* scales as *a*^−*p*^, where *a* is the lattice constant and *p* ≫ 1 for nearest neighbors. If there is a sinusoidal lattice modulation of amplitude *δa*, the linear corrections will average out over the stripes, but there will be a *finite quadratic correction*, *δt*_*x*_ ~ (*δa*)^2^. For vertical stripes, which have no bond length modulation along *y* (the direction along the stripe), we have on the average *t*_*x*_ > *t*_*y*_, and the wave couples quadratically to a *q* = 0 shear mode. Similarly, if the stripes fluctuate in time or space (due to impurity pinning), then *δa* = (*δa*_0_)cos(*q*_*x*_*x*) will also average to zero, but since 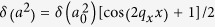, this will not be the case for the shear contribution at *q* = 0. This in turn leads to a *nematic electronic order*: the hole doping is inequivalent on the in-plane *x* and *y* oxygens, as recently found^5^ in STM studies of Bi2212.

Note that even conventional CDW systems tend to have anomalously small correlation lengths^22^,^37^. While the reasons for this are not well understood, impurity effects are likely to be important^38^. In the cuprates, near two-dimensionality means that impurity effects^39^,^40^ and quantum fluctuations^41^ will play a larger role, and the competition with superconductivity will tend to limit the correlation length^32^,^42^. A strong role of impurities in the cuprates is suggested by the fact that correlation lengths in YBCO are ~20 lattice constants^32^, but only ≃8 lattice constants in Bi2212, which is substantially more disordered^24^,^43^,^44^.

In contrast, the magnetic order is less sensitive to impurities. For example, in electron-doped and lightly hole-doped cuprates, the dominant order is found to be (*π*, *π*) antiferromagnetism (AFM)^45^, which does not couple to charge order and hence it is insensitive to charged impurities. In fact, in electron-doped cuprates, long-range order persists to high doping levels, and the divergence in magnetic correlation length is only cut-off when superconductivity emerges^42^. Similarly, in LSCO the dominant instability appears to be magnetic, with the charge order playing a secondary role. In this case also impurities may disrupt the long-wavelength stripe order and support the formation of short stripe segments, which eventually undergo a transition towards a ferronematic state^46^. On the other hand, the structural transition towards a low temperature tetragonal (LTT) phase in La_2−*x*_Ba_*x*_CuO_4_ (or LSCO, codoped with Eu or Nd) is able to pin regular stripes and correspondingly induce a state with longer-range charge and magnetic order, up to ~70 lattice constants.

The nematic order may thus be viewed as an *emergent* phenomenon. The primary instability is to a CDW order, which is unstable to the presence of charged impurities in two dimensions^39^,^40^. On the other hand, there also is a secondary coupling to a strain induced long-range distortion, which is less sensitive to impurities^47^. The general Landau-Ginzburg effective Hamiltonian for modeling the strain-density wave interaction is:


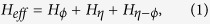


where the density-wave (DW) order is characterized by a charge density modulation given by^48^


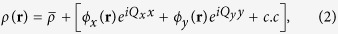


and *ϕ*_*x*_ and *ϕ*_*y*_ are the competing density waves in two orthogonal directions. Then


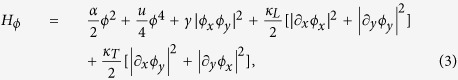


with 
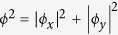
. The strain Hamiltonian is^49^,^50^


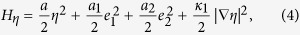


with DW-strain coupling


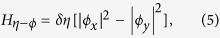


where the strain components are 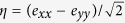, 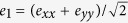, *e*_2_ = *e*_*xy*_. Here, we have integrated out the electronic degrees of freedom, noting, e.g., that *t*_*x*_ − *t*_*y*_ couples linearly with *η*. [In the present one-band model, the effect of strain on the oxygens, which leads to nematic order, is implicit.]

In order to compare with STM results, we assume the density wave above to be ordered (*α* < 0) but strongly pinned, so that it can be represented as a lattice of squares of size *L* × *L*, in each of which the stripe order lies along the *x*- or *y*-axis. The stripe order of each domain can then be reduced to an Ising variable^51^, *σ* [+ for *ϕ*_*x*_, − for *ϕ*_*y*_], and *H*_*ϕ*_ becomes


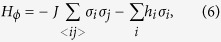


where *σ*_*i*_ denotes the average over a patch, the first sum is over the nearest neighbors (<*ij*>) in the square lattice of patches, and *h*_*i*_ is a random variable over the range (−*h*_0_, *h*_0_). The strain remains a continuous variable, but it is now defined over the patches. Since only the deviatoric strain *η* couples to the stripes, the bulk dilational (*e*_1_) and shear (*e*_2_) strains can be eliminated from the problem. However, there is a compatibility condition relating the strain components, to satisfy Saint-Venant’s principle, which leads to a long-range interaction of the *η* strains^50^,^52^,


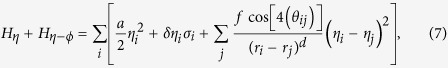


where we have used a renormalized *δ*, and the fact that *σ*_*i*_ is an Ising variable, and *θ*_*ij*_ is the angle between grains *i* and *j* measured with respect to the Cu-O bond direction.

## Results and Discussion

**Figure 1:**
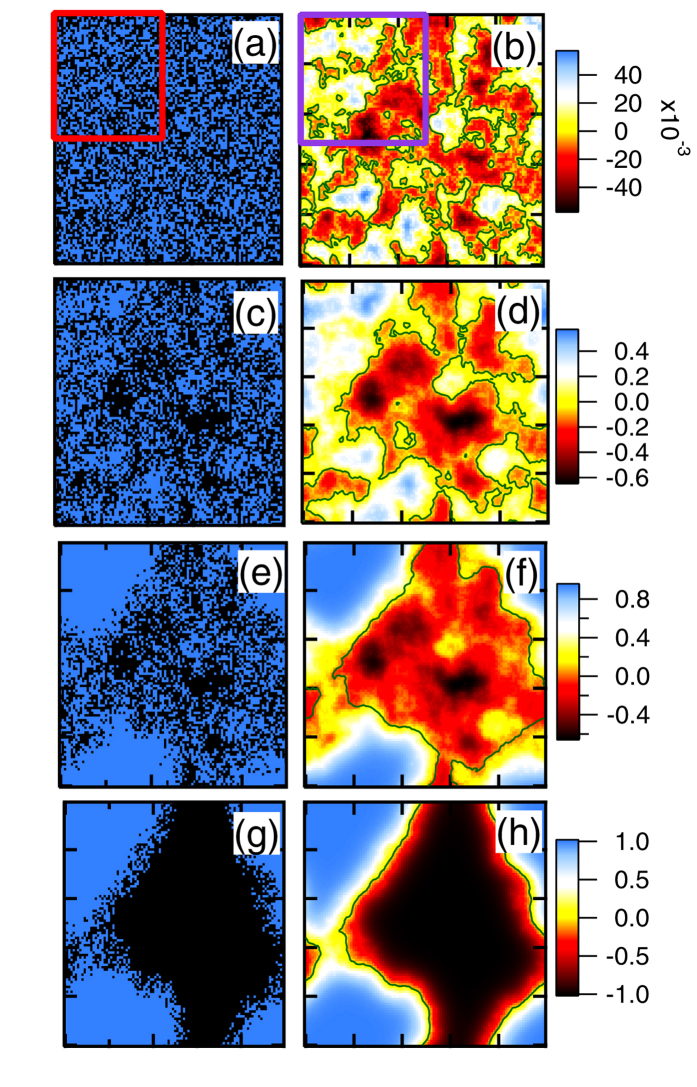
Co-evolution of DW and shear [or nematic] orders in Bi2201. Left: Maps of 100 × 100 DW domains. Black dots refer to *x*-directed stripes (*σ*_*i*_ = +1) and blue dots to *y*-directed stripes (*σ*_*i*_ = −1). Right: Corresponding strain fields (*η*) with values given by the color bar. Different rows correspond to *δ* = 0.2 (**a**,**b**), 0.94 (**c**,**d**), 0.945 (**e**,**f**), and 1.0 (**g**,**h**). The *η* = 0 contour is indicated by solid black lines in frames (**b**,**d**,**f**,**h**). Calculations assume periodic boundary conditions.

Numerical results for the DW *σ*_*i*_ and strain *η*_*i*_ fields are shown in Fig. 1, where we have taken, in units of *a* = 1, *h*_0_ = 0.7, *f*/*L*^2^ = 8 [*L* is the patch size], *J* = 0 [results are not sensitive to small values of *J*], and allowed *δ* to vary. Also, we assume a surface patch network (*d* = 2), but results for a bulk array (*d* = 3) are similar. The small value of *a* with respect to *f*/*L*^2^ suggests that the strain field is nearly unstable. This is to be expected, since this strain couples strongly to the splitting of the Van Hove singularity (VHS) peak.

**Figure 2:**
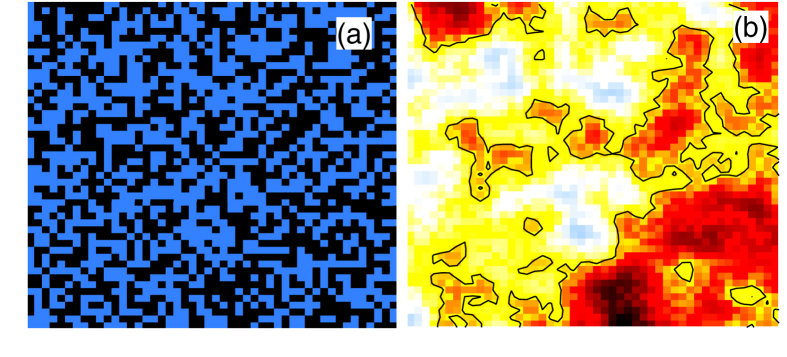
Blow-up of highlighted areas of frames (**a**) and (**b**) of Fig. 1.

Figure 1 shows how the random patches of DW order generate strain fields, and how the strain and DW coherence lengths evolve as the DW-strain coupling is increased. Frames (a,c,e,g) show the DW domains for four increasing values of *δ*, while frames (b,d,f,h) show the corresponding strain fields. Figure 2(a,b) show blow-ups of the highlighted portions of Fig. 1(a,b). In frame (a) each square represents a patch of stripe order aligned along the horizontal (*x*) direction [black squares] or the vertical (*y*) direction [blue squares]. To match STM experiments, each square should be assumed to be *L* ~ 60 Å on a side.

The model calculation of Fig. 1 allows us to probe the strain-DW interaction into the strong coupling regime, see frames (c–h). The boundary separating *η* > 0 and *η* < 0 strains (black lines) is seen to change little with increasing *δ*: the main changes are that small enclosed islands of reversed strain get filled in, the boundary gets smoother (less fractal), and the strain in the large domains gets more uniform. At the same time, the strain tends to align the DW order, leading to a clear excess of one sign of DW in a given strain domain, an effect which has not been reported in STM. When *δ* is very large, frames (e–h), the strain fields are strongly coupled to the DWs, and both display long-range coherence despite the random pinning. Since calculations preserve the net pseudospin, ∑_*j*_*σ*_*j*_, this implies that all up pseudospins segregate into one domain, while all down pseudospins segregate into another domain.

However, the experimentally relevant regime in the cuprates corresponds to small coupling, frames (a) and (b)^5^. While the stripe distribution in (a) is essentially indistinguishable from the pattern in the absence of strain coupling, the strains are already correlated over a substantial distance. Moreover, as *δ* is further reduced, the strain pattern remains unchanged, while the magnitude of the strains decreases, ultimately vanishing as *δ* → 0. In this low-coupling regime, the shear direction is determined by a collective pinning where there is a slight excess of one sign of DW over the other.

Recently, Phillabaum *et al.*^7^ analyzed scaling properties of the nematic domains seen in STM by digitizing the data to just *x*- vs *y*-domains, neglecting variations in the strength of nematic order. Hence, their Fig. 2b can be directly compared with our Fig. 2(b) with respect to the black-line contour signaling the separation into *x*- and *y*-domains. Notably, they find a large percolating region with embedded domains with flipped order, quite similar to our Fig. 2(b). When the strain-DW coupling has increased to *δ* = 0.94, our Fig. 1(d) shows that most of the minority-pseudospin inclusions have been eliminated, leading to a substantially different pattern. Hence, our model correctly captures the large disparity between DW and nematic correlation lengths, even though the DW is the primary order parameter.

After our model was proposed^22^, experimental evidence has been obtained which appears to support the presence of an intimate connection between the CDW and *q* = 0 charge orders^53^, which is a key assumption underlying our model. Notably, the nematic domains in our model are quite large so that within each domain there are local atomic displacements in the BiO layers, which might be observable via STM. Several alternative models have also been proposed^38^,^53^, but it is not clear why the nematic and DW correlation lengths are so different in these other models.

While our model has been phrased in terms of single-*q* CDWs (i.e., CDWs running along the *x*- or *y*-axis in a single domain), there is considerable discussion as to whether there might be two-*q* CDWs – i.e., overlapping *x*- and *y*-CDWs, sometimes referred to as crossed-stripe or, mistakenly, ‘checkerboard’ domains. While such a model can explain the high-magnetic field Fermi surface proposed based on the observed quantum oscillations^54^, it is not clear that this phase persists down to zero field^55^. Recent experiments have failed to find (*q*, *q*) diffraction peaks characteristic of coherent crossed-stripes^56^, while STM studies find that domains of predominantly single-*q* CDWs are universal in cuprates^57^. We stress that our model is not restricted to single-*q* domains, but remains valid as long as most domains have *C*_2_ symmetry (e.g., more *x*- than *y*-DWs), as found in YBCO^58^.

## Conclusions

In conclusion, our study indicates that while charge order in the cuprates is unstable with respect to quenched disorder, it has a quadratic coupling to a *q* = 0 shear mode, which may be the anomalous ‘nematic’ phase seen in STM experiments. Similar effects seem to be at play in many other materials, pointing to the important role of strains more generally^8^,^59^. It would be interesting to explore possible connections between the present model of the *q* = 0 charge order seen in STM studies^5^ and the *q* = 0 magnetic order revealed by neutron scattering experiments^60^,^61^,^62^,^63^. The observation of the latter in LSCO^64^, where dynamical stripes are evidenced by incommensurate magnetic fluctuations, suggests that a similar physics may underlie both phases. Indeed, correlations have been reported recently between the *q* = 0 magnetic order and strong *q* ≠ 0 fluctuations^65^,^66^. If the CDW phase provides an origin for the time-reversal symmetry breaking^67^, then a strain-driven model could perhaps also explain the large associated correlation length.

## Additional Information

**How to cite this article**: Markiewicz, R. S. *et al.* Short range smectic order driving long range nematic order: example of cuprates. *Sci. Rep.* **6**, 19678; doi: 10.1038/srep19678 (2016).
